# Stable Iodine Intake and Thyroid Screening Outcomes After the Fukushima Nuclear Disaster: An Observational Study

**DOI:** 10.1210/clinem/dgaf312

**Published:** 2025-05-29

**Authors:** Yoshitaka Nishikawa, Fumiya Oguro, Chiaki Suzuki, Yurie Kobashi, Naomi Ito, Yoshimitsu Takahashi, Takeo Nakayama, Aya Goto, Masaharu Tsubokura

**Affiliations:** Department of Internal Medicine, Hirata Central Hospital, Hirata, Ishikawa-gun, Fukushima 963-8202, Japan; Department of Health Informatics, Graduate School of Medicine and School of Public Health, Kyoto University, Sakyo-ku, Kyoto 606-8501, Japan; Takemi Program in International Health, Harvard T.H. Chan School of Public Health, Boston, MA 02115, USA; Department of Internal Medicine, Hirata Central Hospital, Hirata, Ishikawa-gun, Fukushima 963-8202, Japan; Department of Thyroid Surgery, Hirata Central Hospital, Hirata, Ishikawa-gun, Fukushima 963-8202, Japan; Department of Early Clinical Development, Kyoto University Graduate School of Medicine, Sakyo-ku, Kyoto 606-8507, Japan; Department of Internal Medicine, Hirata Central Hospital, Hirata, Ishikawa-gun, Fukushima 963-8202, Japan; Department of Radiation Health Management, Fukushima Medical University School of Medicine, Fukushima City, Fukushima 960-1295, Japan; Department of Radiation Health Management, Fukushima Medical University School of Medicine, Fukushima City, Fukushima 960-1295, Japan; Department of Implementation Science in Public Health, Kyoto University School of Public Health, Sakyo-ku, Kyoto 606-8501, Japan; Department of Health Informatics, Graduate School of Medicine and School of Public Health, Kyoto University, Sakyo-ku, Kyoto 606-8501, Japan; Takemi Program in International Health, Harvard T.H. Chan School of Public Health, Boston, MA 02115, USA; Center for Integrated Sciences and Humanities, Fukushima Medical University, Fukushima City, Fukushima 960-1295, Japan; Department of Internal Medicine, Hirata Central Hospital, Hirata, Ishikawa-gun, Fukushima 963-8202, Japan; Department of Radiation Health Management, Fukushima Medical University School of Medicine, Fukushima City, Fukushima 960-1295, Japan

**Keywords:** iodine isotopes, thyroid neoplasm, Fukushima nuclear accident, disaster medicine, case study

## Abstract

**Context:**

Stable iodine intake is crucial in preventing thyroid cancer after radiological emergencies; however, the association between stable iodine intake and thyroid outcomes in children after the Fukushima Daiichi Nuclear Power Plant (FDNPP) accident remains unclear.

**Objective:**

To describe thyroid screening outcomes and investigate the association between stable iodine intake and those outcomes in children after the FDNPP accident.

**Methods:**

This was an observational study based on data from the regional thyroid screening program conducted by the Research Institute of Radiation Safety for Disaster Recovery Support in Fukushima, Japan. Participants were children born between April 1998 and March 2011 in Miharu Town (n = 1974), where stable iodine intake was implemented during the FDNPP accident. The association between stable iodine intake and thyroid ultrasound results was examined using logistic regression analysis. Coarsened exact matching was used to balance sex and age.

**Results:**

Among the participants, 1095 (55.5%) consumed stable iodine, whereas 879 (44.5%) did not. In the age- and sex-matched group of 1952 children (1088 in the intake and 864 in non-intake group), no association was observed between stable iodine intake and thyroid screening results indicating the need for a detailed examination (odds ratio: 0.839; 95% CI: 0.393-1.8, *P* = .647). The volume and parenchymal heterogeneity were not different between these groups.

**Conclusion:**

Stable iodine intake was not associated with thyroid ultrasound screening results, probably because of the low radiation doses following the FDNPP accident. Parenchymal heterogeneity and thyroid volume were similar, supporting the conclusion that the adverse effects of single-dose stable iodine are minimal.

Thyroid cancer is a major concern in the aftermath of a radiological emergency. Stable iodine intake is crucial for preventing thyroid cancer after nuclear emergencies, alongside evacuation, sheltering, and avoiding contaminated food and beverages (1, 2). The administration of stable iodine saturates the thyroid gland, preventing it from absorbing radioactive iodine and internal radiation exposure (1). Therefore, stable iodine intake is recommended for children, adolescents, and pregnant women (1). Iodine blocking was performed in neonates and adults in Poland following the Chernobyl accident (3, 4). However, information on thyroid screening results and stable iodine intake in nuclear disaster cases is limited.

The Fukushima Daiichi Nuclear Power Plant (FDNPP) accident followed the Great East Japan Earthquake and tsunami on March 11, 2011. Local residents were inadvertently exposed to radioactive materials following their release (5, 6). As there were difficulties in obtaining, analyzing, and communicating accurate information, the intake of stable iodine was not directly instructed by the Japanese government (7). However, 7 local governments distributed stable iodine to residents, and 4 instructed them on its intake (8). This was done in coordination with public health nurses and pharmacists when doctors were unavailable. To follow up on the thyroid health of local residents, the Research Institute of Radiation Safety for Disaster Recovery Support (RSDRS) has initiated voluntary thyroid screening since 2012 in collaboration with local governments (9, 10), slightly earlier than the Fukushima Health Management Survey (FHMS) (11). This program has been collecting clinical data and questionnaire-based information, including data on the use of stable iodine. The intake rate of stable iodine can be examined using the RSDRS thyroid screening database. Nonetheless, little is known about the relationship between stable iodine prophylaxis and thyroid screening outcomes after the FDNPP accident.

This study aimed to describe thyroid screening outcomes and examine the association between stable iodine intake and these outcomes among children after the FDNPP accident. By examining these outcomes, this study provides insights into the effects of iodine prophylaxis on the thyroid gland following an FDNPP accident.

## Methods

### Study Design, Setting, and Participants

**Figure 1.**
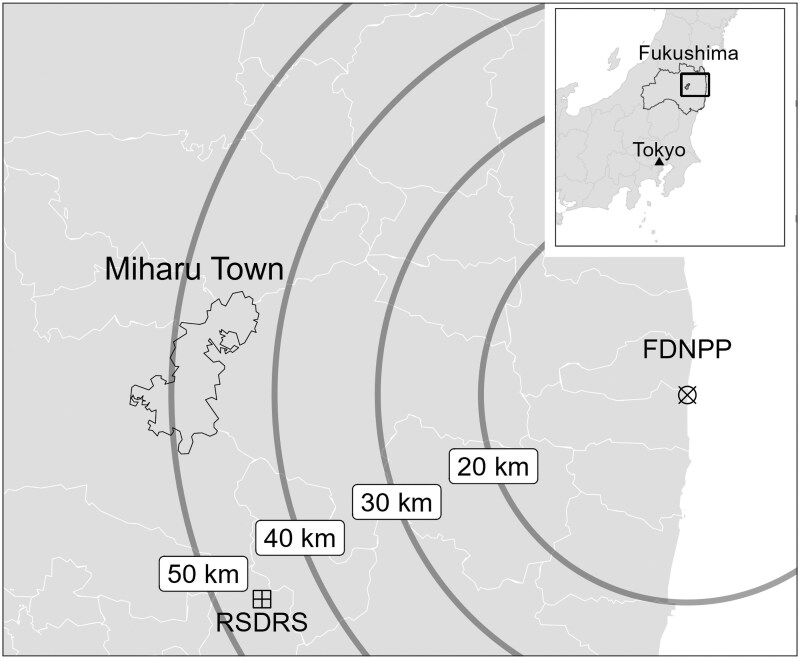
Geographical locations of Miharu Town, Fukushima Daiichi Nuclear Power Plant (FDNPP), and Research Institute of Radiation Safety for Disaster Recovery Support (RSDRS).

Data from a thyroid screening program at the RSDRS in Fukushima Prefecture, Japan, were used in this observational study. Figure 1 shows the geographic locations of the RSDRS, FDNPP, and Miharu Town. The founder of the healthcare corporation Seireikai Group—which owns Hirata Central Hospital, a privately operated 143-bed hospital—established the public interest incorporated foundation RSDRS in response to the FDNPP accident. The participants in this screening program were children born between April 1998 and March 2012 in Miharu Town, which implemented stable iodine intake after the FDNPP accident (10). They underwent thyroid screening at the RSDRS between April 2013 and March 2023 (Japanese fiscal year 2022). Miharu Town implemented stable iodine intake at the time of the FDNPP accident (8). Participants born after March 15, 2011 (the date of the Unit 4 explosion at the FDNPP) (12) or those who did not respond to the questionnaire regarding stable iodine intake were excluded.

### Thyroid Screening at RSDRS

The RSDRS has conducted voluntary thyroid screening since 2012. This screening program was performed separately from the FHMS, which followed the general population of Fukushima Prefecture (13). Differences were observed between these 2 screening programs, particularly in terms of participants and timing. Thyroid screening at the RSDRS was implemented in collaboration with sub-prefectural municipalities before the FHMS. With children born in the year of the disaster as the potentially final cohort, outreach attempts to the municipality may come to a close as the years pass. The RSDRS has been transitioning to a service model that focuses on individuals who voluntarily seek screening.

### Self-Administered Questionnaire

All guardians of the participating children were asked to complete a self-administered questionnaire before thyroid screening at the RSDRS. The questionnaire asked about participants' post-disaster stable iodine intake. All questionnaires were gathered using the RSDRS.

### Data and Variables

The data extracted from the thyroid screening database included participants' age at the time of screening, their sex, and whether they took stable iodine orally after the disaster. Multiple imputations were considered if any variables had missing data.

### Thyroid Screening Outcomes

The primary outcome was the thyroid screening results. Moreover, thyroid volume and parenchymal heterogeneity (+ or −) were retrieved. Thyroid screening results were classified into 4 categories (A1, A2, B, and C):

A1: No nodules or cysts were observed in the thyroid gland. The thyroid gland was considered normal.

A2: Although nodules or cysts are observed, they are benign or of little concern. Cysts and nodules are 20.0 mm or less and 5.0 mm or less in diameter, respectively. This is considered the normal state of the thyroid.

B: Nodules greater than 5.1 mm in diameter and/or cysts > 20.1 mm. Alternatively, nodules or cysts with suspicious characteristics, regardless of size, were classified as B. These participants are recommended for a more detailed procedure known as a “*confirmatory examination*.”

C: Participants require immediate detailed investigation due to the possibility of malignancy, regardless of the nodule or cyst size.

Subsequently, participants classified as B and C are recommended for detailed examinations. The thyroid ultrasound results were evaluated by a board-certified otorhinolaryngologist from the Japanese Society of Otorhinolaryngology-Head and Neck Surgery (C.S.).

### Analytical Methods

Continuous variables are expressed as means and SD. Participants were classified into 2 groups based on their characteristics: intake and no-intake groups. A *t* test was used for analyzing continuous variables, and a chi-squared test was used for categorical variables. The frequency of further examination needed (B or C) was compared between the participants of this study and the same age group in the FHMS (14).

The cohort was matched using coarsened exact matching (CEM) to estimate the average treatment effect on the treated group, that is, the intake group. The matched variables were the age of participants at the time of screening (6-15 years, with a bin width of 1 year), the time of the disaster (2-12 years, with a bin width of 2 years), and sex. The balance between groups was examined using the standardized mean difference (SMD). An SMD of less than 0.1 was interpreted as balanced. To analyze the association between stable iodine intake and the need for a detailed thyroid examination, Firth's bias-reduced logistic regression was conducted using the logistf package in R. The model was adjusted for sex and age at the time of the disaster, accommodating the possibly low incidence of outcomes to provide reasonable estimates despite potential separation issues (15, 16). Statistical significance was set at 0.05. All analyses were performed using R version 4.4.1 (http://www.r-project.org).

### Ethical Considerations

This study was approved by the ethics boards of Hirata Central Hospital (2017-0321-2), Kyoto University (R1459), and Fukushima Medical University (30180). Written informed consent was obtained from the guardians of all participants.

## Results

**Figure 2.**
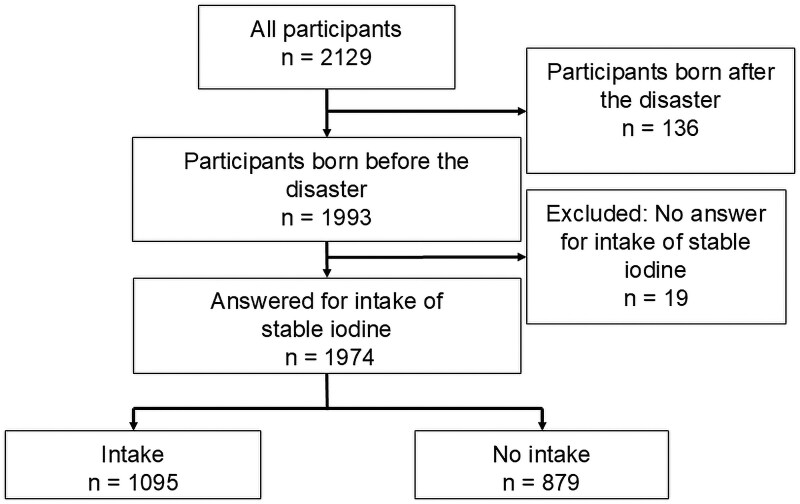
Participants flow chart.

The total number of participants in this study was 2129. Among these, we excluded 136 individuals born after the disaster. Additionally, 19 participants who did not provide information regarding their stable iodine intake were excluded. A total of 1974 children were included in this study (Fig. 2).

Table 1 presents the characteristics and thyroid ultrasound results of the original and matched cohorts divided by stable iodine intake. Among these participants, 1095 (55.5%) reported stable iodine intake, while 879 (44.5%) did not. No missing data exist for any of the listed variables.

**Table 1.** Participants' characteristics and thyroid ultrasound results in the original and matched cohorts, stratified by participants' stable iodine intake

	Original cohort				Matched cohort		
	Overall	Intake	No intake		Intake	No intake	
N	1974	1095	879	*P* value	1088	864	*P* value
***Participants characteristics***							
**Sex**				.575			>.999
** Female**	929 (47.1%)	522 (47.7%)	407 (46.3%)		504 (46.3%)	400 (46.3%)	
** Male**	1045 (52.9%)	573 (52.3%)	472 (53.7%)		584 (53.7%)	464 (53.7%)	
**Age at test (years)**	13.19 (1.57)	13.42 (1.46)	12.91 (1.65)	<.001	12.98 (1.56)	12.98 (1.56)	>.999
**Years since disaster**	6.90 (3.00)	6.45 (2.86)	7.47 (3.08)	<.001	7.50 (3.09)	7.51 (3.09)	.994
**Age at disaster (years)**	6.29 (3.70)	6.97 (3.52)	5.44 (3.74)	<.001	5.48 (3.75)	5.48 (3.75)	.995
***Thyroid ultrasound results***							
**Final result**				.964			.957
** A1**	332 (16.8%)	180 (16.4%)	152 (17.3%)		888 (81.6%)	702 (81.3%)	
** A2**	1613 (81.7%)	899 (82.1%)	714 (81.2%)		186 (17.1%)	149 (17.2%)	
** B**	27 (1.4%)	15 (1.4%)	12 (1.4%)		12 (1.1%)	12 (1.4%)	
** C**	2 (0.1%)	1 (0.1%)	1 (0.1%)		2 (0.2%)	1 (0.1%)	
**Volume (mm^3^)**	9559.14 (3187.13)	9606.04 (3134.37)	9500.71 (3252.51)	.467	9303.56 (3108.64)	9578.40 (3220.46)	.081
**Parenchymal heterogeneity**				.697			.212
** -**	1865 (94%)	1037 (95%)	828 (94%)		1039 (96%)	814 (94%)	
** +**	109 (5.5%)	58 (5.3%)	51 (5.8%)		49 (4.5%)	50 (5.8%)	

Matched variables were sex, age at test, and age at disaster. Data are shown as n (%); mean (SD).

In the original cohort, 47.1% of the participants were female, and 52.9% were male. The average age at the time of the test was 13.19 years (SD 1.57). On average, participants experienced the disaster 6.90 years before, with those in the iodine intake group having experienced it more recently (mean 6.45 years) compared to the non-intake group (mean 7.47 years).

Regarding the thyroid ultrasound results, most participants were classified as A2 (81.7%), followed by A1 (16.8%), B (1.4%), and C (0.1%). In both the intake and non-intake groups, 1.5% of the children required detailed examination. The average thyroid volume (mm^3^) was 9559.14 (SD 3187.13). Moreover, the proportions of parenchymal heterogeneity were similar between the groups. The proportion of further examinations needed in this study was 1.5%, compared to 1.1% in the FHMS (95% CI: −0.12 to 0.84; *P* value = .141).

In the matched cohort, 1952 participants (1088 in the intake group and 864 in the no-intake group) were matched using CEM for a balanced group comparison. The ultrasound thyroid screening results showed that similar proportions of detailed examinations were required: (1.3% in the intake group and 1.5% in the no-intake group).

Table 2 presents the results of Firth's bias-reduced logistic regression, which examined the association between stable iodine intake and the need for a detailed examination, adjusting for sex and age at the time of the disaster. In the original cohort, the odds ratio for stable iodine intake was 0.793 (95% CI: 0.38 to 1.684, *P* value = .539). For the cohort matched by CEM, Firth's bias-reduced logistic regression adjusted for sex and age at the time of the disaster, the odds ratio for stable iodine intake was 0.839 (95% CI: 0.393 to 1.8, *P* = .647). These results suggest that stable iodine intake had no observable effect on the likelihood of requiring further thyroid evaluation in this cohort.

**Table 2.** Firth's bias-reduced logistic regression examined the association between stable iodine intake and the need for detailed examination, adjusting for sex and age during the disaster

Cohort	Variables	Variable role	Reference	Odds ratio	95% CI	*P* value
**Original cohort**	Stable iodine intake	Variable of interest	No intake	0.793	(0.38, 1.684)	.539
	Sex	Covariate	Female	0.345	(0.146, 0.741)	.006
	Age at disaster (years)	Covariate (continuous)	—	1.158	(1.042, 1.298)	.006
**Matched cohort**	Stable iodine intake	Variable of interest	No intake	0.839	(0.393, 1.8)	.647
	Sex	Covariate	Female	0.369	(0.154, 0.809)	.012
	Age at disaster (years)	Covariate (continuous)	—	1.188	(1.072, 1.329)	.001

## Discussion

This study investigates the association between stable iodine intake and thyroid screening outcomes among children following the FDNPP accident. No association was observed between stable iodine intake and thyroid screening results in children after the FDNPP accident. The rate of further examination needed was similar in this population compared with that of the Fukushima Health Management Survey (14, 17, 18). The lack of a significant relationship may be due to radiation doses being too low to affect the thyroid gland (19). According to the United Nations Scientific Committee on the Effects of Atomic Radiation (UNSCEAR) 2020/2021 report, estimated thyroid doses for young children in most areas outside the evacuation zone were below 10 mGy. In Miharu Town, the dose was even lower, under 5 mGy (19). In addition, a previous study showed that usual iodine intake was sufficient in the same population (9). At such low levels of exposure and sufficient level of intake, any protective effect of iodine would be difficult to detect, and our results should not be interpreted as contradicting the known benefits in high-exposure settings. These results should also not be viewed as a critique of decisions made by local municipalities during thyroid protection measures, as their choices were based on the information available at the time of the disaster and the unforeseeable trend in exposure levels. Nevertheless, these findings might guide decisions regarding stable iodine implementation in future nuclear emergencies.

Parenchymal heterogeneity and thyroid volume on ultrasound screening showed no significant differences between the intake and non-intake groups. Parenchymal heterogeneity has been associated with diffuse thyroid diseases, including Hashimoto's thyroiditis (20, 21). These findings are consistent with a previous report showing no adverse effects on thyroid function using single-dose stable iodine 7 years after the disaster (22). Limited information during the radiological emergency makes it difficult to determine which counter measures should be taken; however, this study suggests that any harm from stable iodine to the thyroid gland appears to be minimal. Whether the national government issues such instructions immediately after a nuclear disaster depends on the circumstances. This study documented a case in which local municipalities initiated the distribution and administration of iodine tablets. The case of Miharu Town may serve as a valuable lesson for the future dissemination and implementation of stable iodine tablets.

Furthermore, identifying appropriate methods for conducting post-disaster investigations is necessary. The circumstances surrounding thyroid examinations have evolved from the early stages of the disasters to the present. This study focused on screenings conducted by the RSDRS, which began earlier than prefectural surveys. This earlier start provided crucial opportunities for residents. However, the implementation of early initiation may differ from that of ongoing service provision. Outreach to municipalities by the RSDRS has been decreasing annually, and the facility is transitioning to offering screenings only to individuals who actively seek them. Thyroid screening programs initiated to alleviate these concerns should not increase anxiety. The thyroid screening provided by the RSDRS offers valuable insights into the role of public interest-incorporated foundations in conducting and phasing out examinations in response to a disaster.

This study has certain limitations. First, the findings were based on self-reported iodine intake. Second, because the number of participants was small, it might have been challenging to examine possible associations. However, we collected data from as many participants as possible, corresponding to the number of births in the town (23). Third, although all participants were registered residents of the town at the time of the accident, we could not confirm their exact physical location during the disaster. Therefore, some uncertainty remains regarding each individual's proximity to the FDNPP. Finally, this study was conducted in one municipality and cannot be generalized to all affected areas where stable iodine intake was provided. The decision was based on both the distance from the FDNPP and the actual radiation dose. However, as 3 other municipalities had mandatory evacuations, it was difficult to conduct surveys on a municipal basis outside of this study. Thus, this study is important for describing the actual circumstances and thyroid outcomes following a radiological emergency.

In conclusion, there was no association between the oral administration of stable iodine and thyroid screening results in children after the FDNPP accident. This was likely due to the relatively low radiation doses after the Fukushima nuclear disaster which affected the thyroid gland. No differences were observed in parenchymal heterogeneity or thyroid volume. This finding supported the adverse effects of single-dose stable iodine on the thyroid gland would be minimal.

## Data Availability

The datasets generated for this study were not publicly available; however, they are available upon reasonable request from the authors, subject to approval from Hirata Central Hospital.

## Abbreviations

CEM: coarsened exact matching
FDNPP: Fukushima Daiichi Nuclear Power Plant
FHMS: Fukushima Health Management Survey
RSDRS: Research Institute of Radiation Safety for Disaster Recovery Support
